# O39c. An innovative device for objective hand disinfection control

**DOI:** 10.1186/1753-6561-5-S6-O91

**Published:** 2011-06-29

**Authors:** T Haidegger, M Nagy, A Lehotsk, L Szilagyi

**Affiliations:** 1 Budapest University of Technology and Economics, Budapest, Hungary

## Introduction / objectives

The importance of hand disinfection has been known since Semmelweis, however, fundamental problems remain existent with hand hygiene. Our team of young engineers created a universal method to teach and verify proper hand disinfection. We target both the health care system and the general public, to educate and control through visualizing, demonstrating and measuring. Motivation: Hand washing is the most eective way to reduce the spread of germs, pathogens and therefore hospital- acquired infection rates. Improved hand hygiene provides better living for people and huge savings for the national health care systems.

## Methods

The idea is to use a non-invasive UV-marked commercial alcoholic hand rub, leave the original hand washing work ow intact, then employ digital image processing to determine clean areas and overlay it on the segmented hand. Images are taken in our prototype box|called Stery-Hand|that gives repeatable and immediate measurement of hand washing quality based on the UV traces of the soap, and visualizes the results in an intuitive form. The modern articial intelligence method employed for clustering (fuzzy c-means) allows us to customize the evaluation and therefore to improve its performance.

## Results

The Stery-Hand was tested in the clinical environment on over a hundred people in three countries: a medical university and a pulmonology institute in Hungary, a county hospital in Romania and a university hospital in Singapore. In addition, it has been widely used at public occasions in Hungary, Austria and France. Beyond providing the comparable numbers of sta compliance, it identied the typical errors, and also revealed several misbehaviors, such as wearing jewelry or articial nails. Overall, the participants did nd it useful and informative, some children even amusing.

## Conclusion

Stery-Hand may become the next breakthrough in medical hand hygiene control, once tightly integrated to the hospital information management system. Improving compliance decreases hospital costs, and also hinders the spread of major epidemics like SARS or H1N1.

## Disclosure of interest

None declared.

## Note

This abstract was presented as O39c.

